# Phage host interactions reveal LPS and OmpA as receptors for two *Erwinia amylovora* phages

**DOI:** 10.1038/s41598-025-15724-z

**Published:** 2025-10-21

**Authors:** Nassereldin Ibrahim, Jason A. McAlister, Jennifer Geddes-McAlister, Antonet M. Svircev, Joel T. Weadge, Hany Anany

**Affiliations:** 1 Agriculture and Agri-Food Canada, Guelph Research and Development Centre, Guelph, ON N1G 5C9 Canada; 2 Department of Biology, Wilfrid Laurier University, Waterloo, ON N2L 3C5 Canada; 3 Molecular and Cellular Biology Department, University of Guelph, Guelph, ON Canada; 4 Agriculture and Agri-Food Canada, Vineland Station, ON L0R 2E0 Canada; 5 Genetic Engineering and Biotechnology Research Institute, University of Sadat City, Sadat City, Egypt; 6 Food Science Department, University of Guelph, Guelph, ON N1G 2W1 Canada

**Keywords:** Fire blight, Phage-carrier system, *Pantoea agglomerans*, *Erwinia* lytic phages, Lipopolysaccharides, Outer membrane protein A, ϕEa21-4, ϕEa46-1-A1, Antimicrobials, Phage biology

## Abstract

*Erwinia amylovora* is the causative agent of fire blight. Resistance to streptomycin, the main antibiotic in fire blight management, has led to an urgent requirement to develop alternative biological control agents, such as the phage-carrier system (PCS). Previous studies have focused on the dynamic interactions between the carrier (*Pantoea agglomerans*), lytic phages, and the pathogen. However, crucial information about phage receptors on these hosts is still lacking. Here, a biochemical approach was used and the phage receptors of two *E. amylovora* phages (ϕEa21-4 and ϕEa46-1-A1) on both hosts, have been identified as LPS and OmpA on *E. amylovora* and OmpA only on *P. agglomerans.* Interestingly, this work uncovered for the first time that amylovoran is tightly attached to the LPS of *E. amylovora*. Confirmation of this interaction and an infection model are presented that have far reaching implications for additional PCS improvement and pathogen-host interaction details.

**Supplementary Information:**

The online version contains supplementary material available at 10.1038/s41598-025-15724-z.

## Introduction

Bacteriophages (phages) are viruses that exclusively infect bacterial cells, shape bacterial genomes, affect physiology and have important impacts on bacterial ecology and nutrient cycles since they represent the most abundant biological entity on earth^1–3^. Lysogenic phages in particular have been noted to play a role in altering the virulence and biofilm formation of host bacteria^4,5^. On the other hand, lytic phages have been used in a wide variety of biotechnology and clinical applications through phage display and phage therapy applications to detect and/or remove specific target bacteria^6,7^. Many forms of phage applications have been developed, with purified phage entities, cocktails, engineered phages or as purified phage-components, to fight against human and plant bacterial pathogen infection for almost a century^8,9^. Much of this development has focused on the start of the phage infection cycle and the crucial step of phage attachment to the host cell by phage tail spike or fiber proteins^10^. Phages recognize their bacterial host with great specificity through distinct receptor(s) on the bacterial host surface. For example, phages can recognize a variety of outer membrane proteins (OMPs), lipopolysaccharide (LPS), exopolysaccharide (EPS), capsular polysaccharides, pili and flagella as host receptors^11–13^. Teichoic acid of Gram-positive bacteria and the LPS terminal residues of Gram-negative bacteria are particularly prominent examples of phage receptors^11^. Interestingly, bacterial host cells have many defense mechanisms to circumvent phage infection, which leads to an ongoing evolution of phage applications and phage receptor identification^14–18^.

Pest management of *E. amylovora* in US and Canadian conventional orchards (non-organic) relies mainly on antibiotics. However, streptomycin resistance has been reported in certain production areas, which in turn has led to the development and use of phages, such as the Agri-Phage commercial product registered in the US and phage-carrier systems (PCS)^18–20^. A recently published protocol for the large-scale production of PCS, involving *P. agglomerans* infected with *E. amylovora* phages, has been developed as a step toward the commercialization of a new biological^21^. In this study, a reconstituted PCS powder of the *P. agglomerans* Pa39-7 strain infected with phage ϕEa21-4 (myovirus, *Kolesnikvirus* Ea214) led to a three log reduction in *E. amylovora* using a pear disc assay and a powder shelf life of four months at 4 °C. Further enhancement of this PCS in other studies focused on understanding the dynamic interactions between *Erwinia* phages, *E. amylovora*, and *P. agglomerans*^22,23^. Roach and colleagues (2013) demonstrated that amylovoran (*rcsB*) mutants of *E. amylovora*, produced productive infections when challenged with *Erwinia* myoviruses (such as ϕEa21-4). In contrast, *Erwinia* podoviruses (such as ϕEa46-1-A1) resulted in no progeny or very low efficiency of plating, ranging from 0 to 0.00026^22^. In contrast, *P*. *agglomerans* Pa39-7 (the phage carrier component in the PCS system) can produce high *Erwinia* myovirus and podovirus progeny populations.

Despite these results, limited information on the specific phage receptors on both bacterial hosts is still lacking. Past studies have identified an *E. amylovora* phage receptor in a T7-like myovirus L1 phage, where amylovoran (the main exopolysaccharides (EPS) component) was the main phage receptor. More recently, it was reported that the lytic podovirus S6 phage possesses a cellulase that helps degrade the cellulose component of the EPS around the targeted bacterial cells to facilitate infection^24–26^. While we are beginning to understand host-phage interactions with *E. amylovora* L1 and S6 phages, the multitude of other *E. amylovora* phage receptors and depolymerases remain uncharacterized despite their increasing incorporation into PCS treatments^27^.

The aim of the present study was to expand our existing knowledge by identifying the phage receptors on two lytic *Erwinia* phages, ϕEa21-4 (myovirus) and ϕEa46-1-A1 (podovirus), that will be a component of lytic phage mixtures incorporated into a PCS. Using biochemical methods, that included DNA ejection, OmpA inhibitor assessment and immunoprecipitation combined with mass spectrometry, we showed that the two *Erwinia* phages demonstrated attachment to OmpA and amylovoran-bound LPS as phage receptors, while using only OmpA on *P. agglomerans.* This work provides a better understanding of the infection dynamics occurring in the PCS and ultimately will assist the further improvement of the efficacy of these PCSs.

## Results

### Lipopolysaccharide-phage receptor analysis

**Fig. 1:**
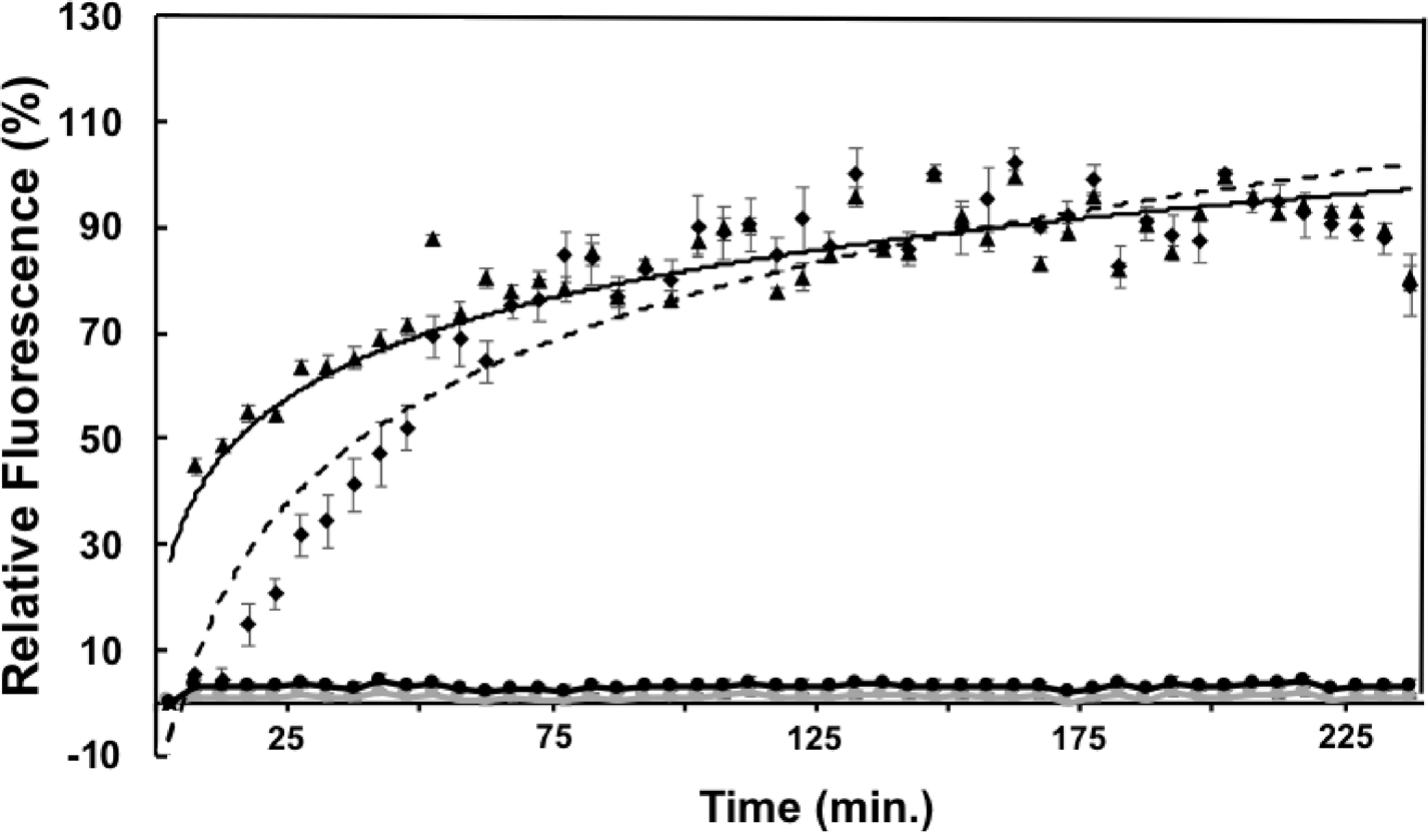
In vitro DNA Ejection from *Erwinia* Phages in the Presence of LPS. To follow DNA ejection at 37 °C, phages were added to a final concentration of 1.0 × 10^8^ PFU/mL with 120 endotoxin units (EU) of LPSs and 1.1 µL of Yo-Pro-1 fluorescent DNA-binding dye. Curves are for the relative fluorescence of Yo-Pro-1-bound DNA ejected from either myovirus ϕEa21-4 phage (◆) or podovirus ϕEa46-1-A1 phage (▲) when incubated with *E. amylovora* D7 LPS. Data fitted to the logarithmic equations for both phages, as shown in solid and dashed lines, respectively. Both phages showed no DNA ejection with *P. agglomerans* Pa39-7 LPS (lines marked with ●). Experiments were performed in three biological replicates and SEs are shown as error bars.

LPS is often reported as a receptor for bacteriophages; this feature was used as an initial step in identifying the phage receptors for phages ϕEa21-4 and ϕEa46-1-A1. Independent incubation of these two phages with purified LPS from *E. amylovora* or *P. agglomerans,* was assessed. Both ϕEa21-4 and ϕEa46-1-A1 recognized the *E. amylovora* LPS and ejected their DNA, which in the presence of Yo-Pro-1 dye (a double stranded DNA binding fluorescent dye) could be detected by monitoring fluorescence changes over time until a maximum intensity was reached (Fig. 1). This maximum intensity indicated full saturation of the exposed DNA with the dye after it was released from the phage head. A parallel approach with *P. agglomerans* LPS led to no fluorescence saturation with either of the two phages (Fig. 1); thereby indicating a lack of phage DNA ejection in the presence of this LPS.

### Carbohydrate analysis of phage treated LPS

To explore the phage effects on *E. amylovora* LPS, carbohydrate analysis was performed on the LPS sample following incubation with ϕEa21-4. After a hydrolysis step, several differences in the glycan peaks were observed between the LPS sample treated with phage compared to the untreated sample (Table 1, Table S1, and Figure S1). These differences are likely attributed to the enzymatic effects of the phage particles on the treated LPS sample that occurred prior to analysis and are evident as either depleted or enriched glycan peaks. Surprisingly, in the phage treated LPS sample, one of the enriched glycan peaks was pyruvate hexose (PyrHex), which is estimated to be approximately 10% of the glycans in this LPS sample. Notably, pyruvate forms a cyclic acetal with the terminal galactose in the amylovoran polymer, the major component of *E. amylovora* EPS, which likely explains the presence of PyrHex, since this modification has not been reported for *E. amylovora* LPS^28,29^. Since amylovoran is generally thought to be a component of the amorphous bacterial cell capsule and not the cells, then it should be removed during the cell-bound LPS purification process. Any remaining contaminant amylovoran would then be expected to be present in both phage treated and untreated samples in approximately equal amounts. However, amylovoran was only observed to be enriched in the phage treated sample (approximately 17% higher). To eliminate the possibility that the amylovoran source came from the phage preparation that was added to the LPS during the phage treatment, the presence of amylovoran was tested using a spectrophotometric method that relies on cetylpyridinum chloride, but amylovoran was not detected^30^. Accordingly, the source of the PyrHex in the phage-treated sample most likely is amylovoran that was present in tight association with the LPS and liberated by the phage. This amylovoran EPS attachment to LPS may be taking place either by cationic bridges, which can be disrupted by EDTA, or through glycosidic bond formation that can be disrupted by specific glycosidase activity.

**Table 1:** Change in glycan pattern of phage treated LPS.

tR (min)	m/z	ID	T/C %	Possible Source
2.78	271.14	Pen	67.6	O-antigen
7.97	371.14	PyrHex	116.9	Amylovoran
8.16	371.14	PyrHex	106.9	Amylovoran
10.48	504.22	HexHexNAc	132.5	LPS/ amylovoran
12.25	609.24	Hex2dHex	114.6	Rhamnose/LPS
14.06	609.24	Hex2dHex	121.4	Rhamnose/LPS
14.06	523.22	Hep2	121.4	LPS
14.35	625.24	Hex3	102.1	LPS/ amylovoran
15.99	755.31	Hex2dHex2	113.8	Rhamnose/LPS

t(R): retention time; m/z: glycan fragment mass/charge; ID, identified glycan; T/C: glycan fragment percentage in the phage treated LPS sample compared to the control, untreated LPS.

**Fig. 2:**
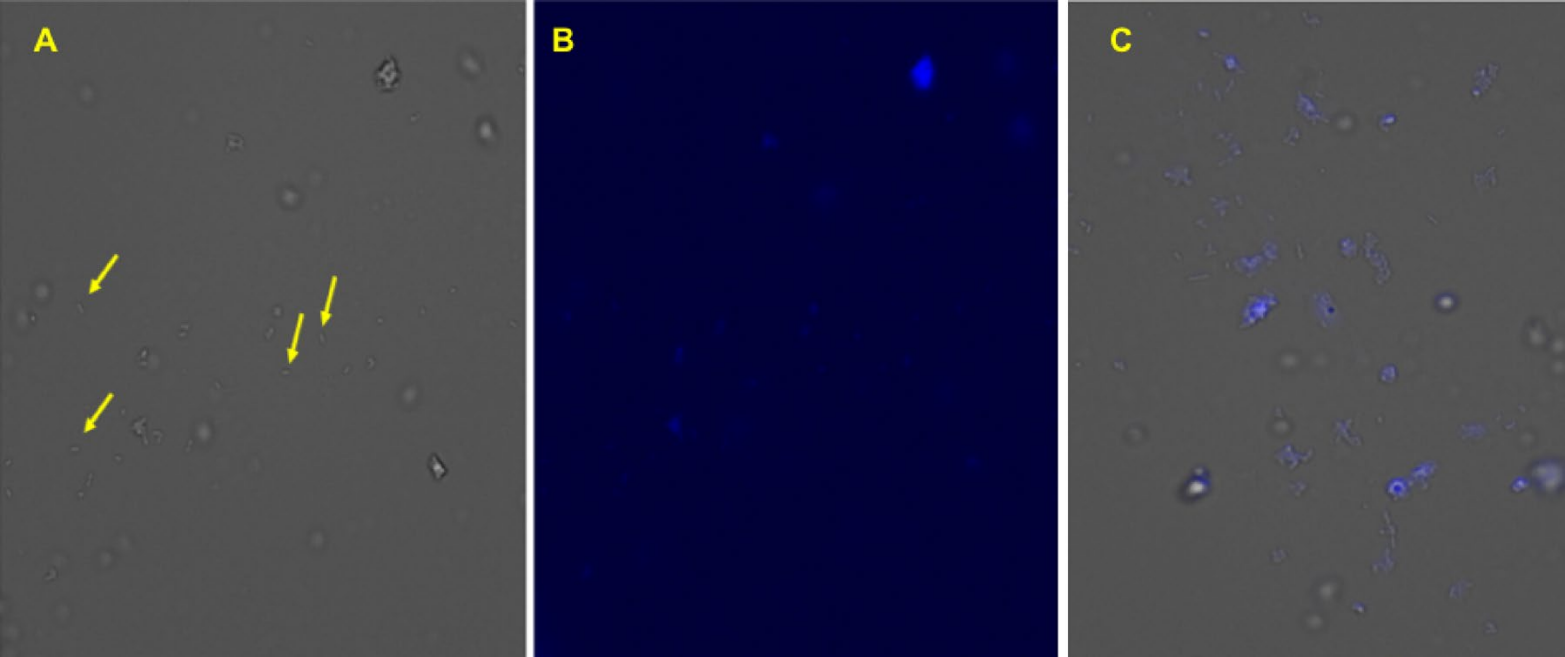
Amylovoran attachment to LPS of *E. amylovora* D7 cells. Mid-log *E. amylovora* D7 cells were incubated with amylovoran-CF®350 and then examined with a fluorescence microscope. Fluorescence could be clearly seen upon attachment of amylovoran-CF®350 to the LPS of *E. amylovora* D7 following extensive washing. *E. amylovora* D7 cells with transmitted light (**A**) or with the DAPI filter (**B**). Yellow arrows indicate the non-fluorescent cells, which emphasizes the the fluorescence is not due to free CF®350 dye or non-specific binding. (**C**) An example of a separate set of superimposed images of transmitted- and DAPI-derived images collected during experimentation for reference. Magnification power 40×.

To confirm the phage glycan-screening results, further analysis of amylovoran attachment to *E. amylovora* LPS was performed using purified amylovoran labelled with a CF®350 fluorescence dye. After dialysis to remove excess dye, CF-labelled amylovoran was filtered and added to growing *E. amylovora* D7 cells. If amylovoran attachment to the LPS does occur through extracellular interactions/enzyme(s), then the labelled-amylovoran should be tightly associated with the bacterial cells. Following treatment with the labelled amylovoran and extensive washing steps, the *E. amylovora* D7 cells fluoresced, confirming LPS association (Fig. 2).

To expand on the microscopy results, the LPS was extracted from the labelled cells, and the fluorescence was measured with a plate reader using CF®350 excitation and emission wavelengths of 347 and 448 nm, respectively. The extracted LPS showed fluorescence indicating the attachment of the CF®350-amylovoran to the LPS of the growing cells (Table 2).

**Table 2:** Fluorescence measurement from CF®350-amylovoran labelled- *E. amylovora* D7 LPS.

Sample	Fluorescence (Ex/Em: 347/448 nm)
Mid-log cells	2307 ± 24
O/N cells	542 ± 14
PBS	59 ± 2

O/N: Overnight culture; PBS: Sod. phosphate buffer.

### Analysis of outer membrane proteins as phage receptors

**Fig. 3:**
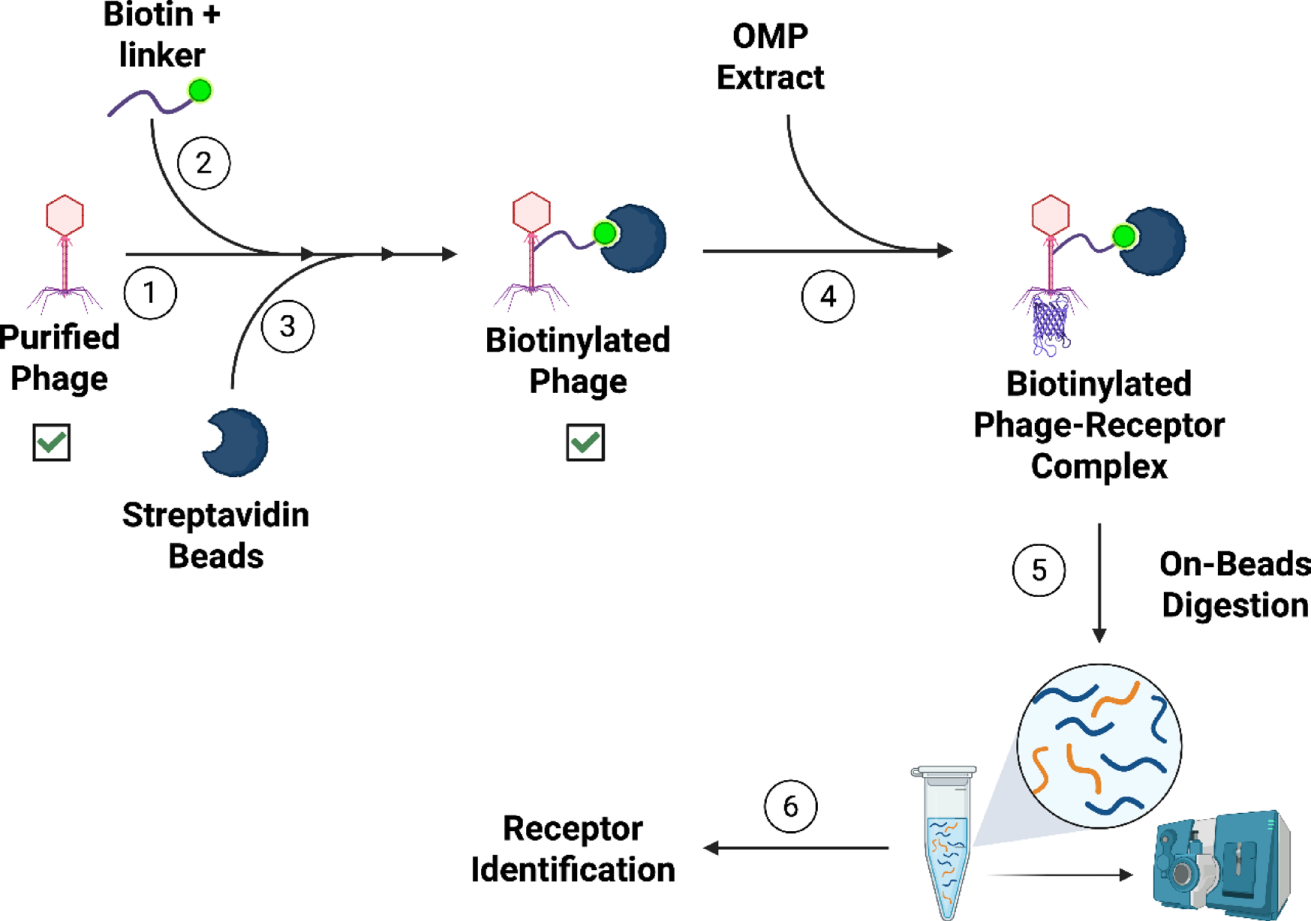
Immunoprecipitation Assay for OMP Receptor Identification. Step 1, the phage was purified by ultracentrifugation. Step 2, the purified phage was biotinylated and biotinylated phage was conjugated with streptavidin beads, as shown in step 3. In step 4, the beads that attached to the biotinylated phage were incubated with OMP extract and the beads were collected by centrifugation. Step 5, the phage with its binding receptor from the OMP extract was subjected to on-bead digestion by trypsin and, in step 6, the resulting peptide was identified by MS/MS analysis to identify the binding receptor. ☑ indicates that the phage was tested to ensure it still can infect *E. amylovora*.

OMPs are frequently identified as receptors for bacteriophages, and we examined the binding of phages ϕEa21-4 and ϕEa46-1-A1 to these proteins. OMP samples were separately purified from *E. amylovora* and *P. agglomerans* cell extracts. Phages ϕEa21-4 and ϕEa46-1-A1 were biotinylated using EZ-Biotin linker and were bound to Streptavidin agarose. Immunoprecipitation (IP) proteomics, including on-bead digestion and MS/MS analysis, were carried out by incubating phage-Streptavidin-agarose with OMP extracts (Fig. 3). Two candidate receptors, OmpA and flagellin, were clearly identified in the OMP extracts from both *E. amylovora* and *P. agglomerans* (Table 3, supplemented data; IP assays.xlsx and Figure S2).

**Table 3:** Identified OMP candidate receptors by immunoprecipitation combined with mass spectrometry.

Candidate receptors	ϕEa46-1		ϕEa21-4		Protein ID	Organism
	No. peptides	Seq. coverage [%]	No. peptides	Seq. coverage [%]		
OmpA	22	56.7	11	29.0	A0A2V1YN43 D4I0C9	*P. agglomerans*/*E. amylovora*
Flagellin	17	74.6	15	48.1	D4HVZ2 D4HYZ7 A0A6I6K319	

**Fig. 4:**
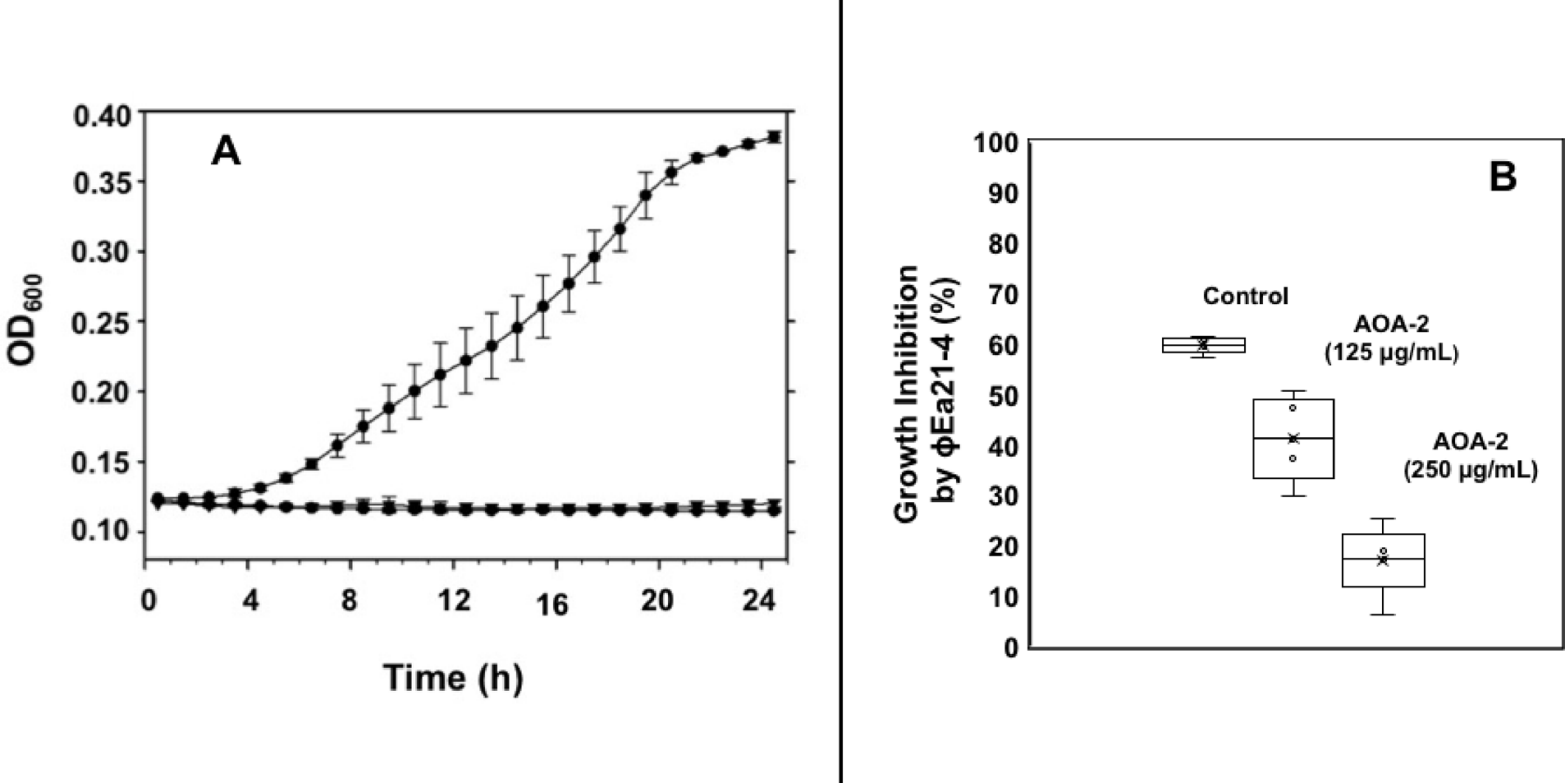
OmpA as principal *E. amylovora* phage receptor. (**A**) Growth curves of the *E. amylovora* 1189 *waaL* mutant (line with solid circles) alone versus in the presence of ϕEa21-4 (line with solid diamonds), or ϕEa46-1-A1 (line with solid inverted triangles) phages. Complete growth inhibition by phages was observed with the *WaaL* mutant. (**B**) ϕEa21-4 infectivity of *E. amylovora* D7 strain at two concentrations of AOA-2 inhibitor [125 and 250 mg/mL] compared to a control without the inhibitor [0 mg/mL]. The presence of OmpA inhibitor mitigates the effect of ϕEa21-4 infectivity on *E. amylovora* D7 strain growth. The standard deviation was calculated from three experimental replicates.

Next, to confirm that OmpA is a phage receptor, two experiments were performed. First, the ϕEa21-4 and ϕEa46-1-A1 phage infectivity of the *E. amylovora* 1189 *waaL* mutant (*E. amylovora* 1189 lacking O-antigen LPS) was compared to the wild type (parent strain) *E. amylovora* 1189 (possessing intact LPS) were assessed^31^. The growth curves of both the mutant and wild type strains were comparable in the absence of phages. However, in the presence of either ϕEa21-4 or ϕEa46-1-A1, the growth of the *Ea*1189 *waaL* mutant was completely inhibited (Fig. 4A). These results indicate that, in the absence of LPS O-antigen, OmpA is fully accessible to the phage and leading to complete inhibition of the growth of the mutant cells.

To further explore the possibility that OmpA is the main receptor, ϕEa21-4 was selected and assessed for infectivity on wild type strain *E. amylovora* D7 in the presence of AoA-2 (a cyclic peptide that blocks and inhibits OmpA)^32^. In the presence of increasing concentrations of AoA-2 peptide, growth inhibition due to phage infection of the wild type strain was alleviated (Fig. 4B). These results demonstrate that blocking of OmpA by AOA-2 was enough to interfere with the phage infection and these results strongly support OmpA as a main receptor for ϕEa21-4.

**Fig. 5:**
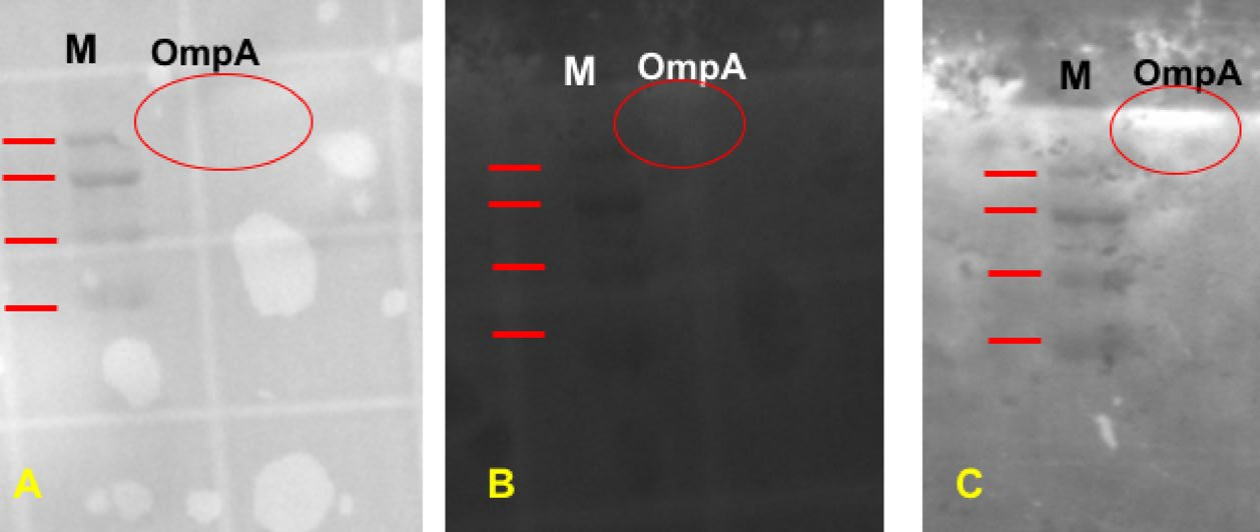
Binding of fluorescent-labelled fEa21-4 phage to *E. amylovora* OmpA following Western blotting. Purified OmpA on a PVDF membrane was imaged using white light (**A**), or UV light (**B**) on a light transmitter. The membrane was re-imaged in UV mode after incubation with fluorescent-labelled ϕEa21-4 and washing with PBS (**C**). The OmpA band was illuminated by UV only after incubation with fluorescent-labelled ϕEa21-4. There is no fluorescence on the molecular weight markers that act as internal negative controls for the experiment.

Lastly, OmpA was also assessed as a phage receptor using Western Blot analysis (Fig. 5A). After incubation of OmpA on the PVDF membrane with CF®350-labelled phage ϕEa21-4, a fluorescent band was observed (Fig. 5C). This fluorescence was absent prior to treatment with the labelled phage (Fig. 5B) and is specific for OmpA (not noted to bind the molecular weight marker controls).

### Assessment of flagellin O-glycosylation as a phage binding site

Flagellin O-glycosylation and LPS have common biosynthetic pathways that can lead to decoration of these structures with the same glycans that phages use as receptors^33^. Given that flagellin was identified in the IP experimental results (Table 3), the possibility that O-glycosylation of this structure was acting as the phage receptor, not the flagellin protein itself, was tested. A sample of the purified *E. amylovora* D7 FliC was deglycosylated by hydrolysis with β1,4-galactosidase and β-N-acetyl-glucosaminidase. The IP assay was repeated with this deglycosylated FliC and the native glycosylated FliC control. In the deglycosylated sample, no FliC fragments were detected, while the glycosylated control was similar to the previous mass spectrometry results with identifiable FliC (Table 4, supplemented data: IP assays.xlsx file). This result supports the theory that flagellin is not a phage receptor; however, it is recruited in the IP assay due to its similar O-glycosylation pattern to that of the LPS from this stain.

**Table 4:** FliC Glycosylation effect from immunoprecipitation assay.

	Deglycosylated Ea FliC	Native Ea FliC
FliC (Q5XPM8_ERWAM)	Not detected	Detected

Ea FliC: *E. amylovora* partially purified FliC protein (Accession #: Q5XPM8_ERWAM).

## Discussion

Continued efforts to control *E. amylovora* infections in apple orchards during open bloom have focused on antibiotics and biologicals in integrated pest management systems^16–18^. A PCS that uses an antagonistic bacterium, *P. agglomerans*, infected with *E. amylovora* phages, was developed and optimized for commercial scale production^21–23^. This phage-mediated biological control system has the capacity to infect and destroy streptomycin resistant and susceptible bacterial populations during open bloom when apple trees are most susceptible to infection by the pathogen^22,23^. The work presented herein complements these previous studies by providing key information on phage receptors for both bacterial hosts, *E. amylovora*, and *P. agglomerans*^22,23^.

Detailed *Erwinia* phage receptor information is limited to a few *Erwinia* phages, including the *E. amylovora* S6 phage that recognizes bacterial cellulose, and L1 and Era103 phages that require amylovoran as a receptor^24–27^. These studies were carried out using a Tn5 transposon library and *amsD* mutants^25,28^. However, it should be noted that other researchers have warned that genetic compensation, feedback mechanisms and the complexity of the biological system requires caution to be taken into account so that all of the receptor possibilities are not overlooked in these mutant screens^34^. Thus, in this study we used a combination of direct biochemical methods and mutants (*waaL*), to identify the *E. amylovora* phage receptor(s) of the ϕEa21-4 (myovirus) and ϕEa46-1-A1 (podoviru*s*) phages. Phages recognize their specific receptor(s) on the host cells and bind to the receptor(s) with high affinity and specificity before subsequently ejecting their genome^35^. The part of the phage machinery that is responsible for recognizing the receptor(s) is either the tail fibers or tail spike protein(s). In general, bacterial LPS, EPS, OMPs and/or flagellin are commonly reported as receptors for bacteriophage. Each of these possible structures were at least partially explored in our analysis of ϕEa21-4 and ϕEa46-1-A1 phage receptors.

The LPS from both *E. amylovora*, and *P. agglomerans* were purified by the Hitchcock and Brown method and used in DNA ejection experiments, with ϕEa21-4 and fEa46-1-A1^36–39^. DNA ejection results demonstrated that *E. amylovora* LPS triggered *Erwinia* phages, ϕEa21-4 and ϕ Ea46-1-A1 to eject their DNA, but no ejection was observed with this assay for *P. agglomerans* LPS (Fig. 1). We propose that the phages recognize the amylovoran attachment to the *Erwinia* LPS, and it is not surprising that *P. agglomerans* LPS, lacking amylovoran and possibly differing in structure, cannot trigger the ejection of these phage genomes and hence no fluorescence was detected. In other studies, host LPS was also able to trigger DNA ejection by P22 and HK620 *E. coli* phages, but the specific epitopes on the LPS were not determined^39,40^.

The DNA ejection process of the two tested phages were noticeably different, as exemplified by their ejection curves (Fig. 1). The podovirus ϕEa46-1-A1 ejected its genome very rapidly, as its ejection curve showed 50% relative fluorescence after just 5–10 min. However, myovirus ϕEa21-4 took almost 45–50 min to reach this point, which likely indicates the differences in the required time for full phage genome ejection. This might be due to the morphological structure differences, since the DNA has to travel through the tail tube in myovirus compared to podovirus, which has no tail tube. Alternatively, ejection times may be varying due to differences in the ejection kinetics. These ejection results are similar to previously published data that showed that phage ϕEa46-1-A1 started its genomic replication after 21 min of infection, has a lytic cycle of 38 min, and burst size of 57 progeny. While ϕEa21-4 starts its genomic replication after 29 min of infection, has a lytic cycle of 98 min, and a burst size of 185 progeny^23^.

In a challenge to identify the exact phage receptor(s) on *E. amylovora* LPS, a ϕEa21-4 phage-treated LPS sample was analyzed by acid hydrolysis and LC–MS/MS and compared to a phage-untreated sample. Surprisingly, the results of the phage treated LPS sample indicated the presence of the namesake *E. amylovora* capsular exopolysaccharide, amylovoran, as the sample was enriched with pyruvate hexose glycan fragments (PyrHex). The presence of these hexoses likely originates from amylovoran degradation by the phage particles and release of the terminal galactose that is pyruvate modified^28,29^. This result is significant as amylovoran is estimated to represent about 17% of the phage treated sample.

Attachment of capsular exopolysaccharide to the LPS by cation bridging or glycosidic bonds is known in some bacteria. *Rhizobium leguminosarum* biovar viciae capsular polysaccharide, which has a similar structure to amylovoran, was found to be tightly attached to *R. leguminosarum* LPS. This association was proposed to be through calcium ion bridging and/or hydrophobic interactions between the capsular hydroxybutanoyl group modifications and LPS^41,42^. The O-antigen and the core oligosaccharide of *R. leguminosarum* are proposed to be involved in this tight attachment between capsular polysaccharides and the bacterial cell surface. LPS-defective mutants of *R. leguminosarum* displayed a 30 to 40% reduction in the capsular polysaccharides when these capsular polysaccharides were extracted from the mutants^43^. Physiologically, the binding/attachment of the capsular polysaccharides to the LPS may have crucial roles for both pathogenic and symbiotic bacteria, as it can help in the bacterial cell attachment to the host and/or help in mitigating the host immune response due to concealment of the more immunogenic bacterial LPS^43^. It is worth mentioning that the *E. amylovora waaL* mutant showed low virulence and more sensitivity to hydrogen peroxide^31^. Accordingly, it was proposed that LPS has a role in virulence and oxidative stress protection during infection. Our present work extends these effects by suggesting that the absence of O-antigen impairs the attachment of the amylovoran to the LPS, which contributes to the overall observed phenotype.

During LPS extraction, the use of a boiling water bath for 30 min and acid hydrolysis prior to MS/MS analysis should lead to liberation of amylovoran from the LPS. The failure to liberate this amylovoran suggests that it is either directly bound to the LPS by glycosidic bonds or very tightly associated by other means. Once the LPS was treated with phage ϕEa21-4, there was an increase in the release of amylovoran products compared to the control LPS samples. Whether the amylovoran is directly bound to the LPS by a glycosidic bond or very tightly associated still requires further biochemical validation. However, an important finding in support of the direct-linkage possibility is that the phage LPS analysis data had an enrichment of HexNAc glycan fragments in the phage treated sample. This coincides with the presence of a H^1^-NMR peak most likely for *N*-acetyl hexose (*e.g.*, *N*-acetyl-galactosamine) noted in the LPS analysis (data not shown). Since the amylovoran has four galactose moieties in its unit structure (in addition to a glucuronic acid residue)^28,29^, one of these galactose residues is most likely in an *N*-acetyl form. According to this proposal, once the phage recognizes the amylovoran linkage to LPS (*i.e.,* the *N*-acetyl-galactosamine-mediated linkage, not just amylovoran alone), it cleaves these glycosidic bonds and liberates the amylovoran residues. This proposed glycosidase activity of phage ϕEa21-4 is not unique, since chitinase activity within two *Erwinia* myovirus Phyllophages AH04 and AH06 has also been noted^44,45^.

**Fig. 6:**
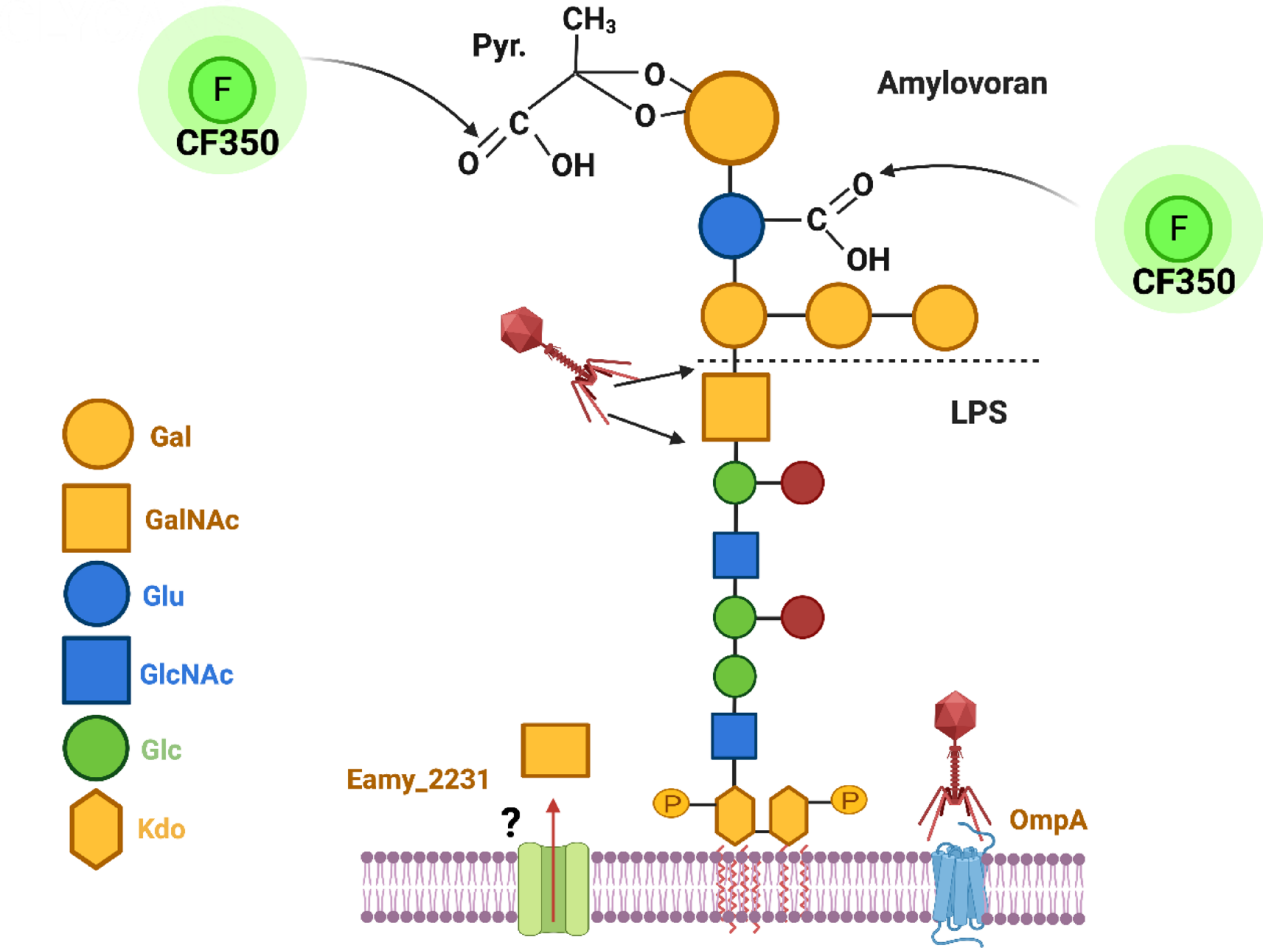
Model for amylovoran and *E. amylovora* LPS. A proposed model for the amylovoran attachment to *E. amylovora* LPS by glycosidic bonds via an *N*-acetyl-galactosamine residue, which may either be from the amylovroan or the LPS (noted positionally in the diagram as a GalNAc on LPS for this diagram). The EAMY_2231 protein may play an important role with GalNAc transfer, either for direct attachment to amylovoran/LPS or through an unknown lipid carrier, that ultimately leads to LPS decorated with amylovoran. During ϕEa21-4 phage infection, the phage breaks the GalNAc-mediated linkage between amylovoran and LPS, via glycosidase activity that was noted in the phage-treated LPS carbohydrate analyses. *E. amylovora* phages can then reach the surface and use OmpA as a main receptor for infection.

To further explore the proposed attachment of amylovoran to LPS, CF^Ò^ Aminooxy fluorescent dye was used to label purified amylovoran from *E. amylovora* D7 strain, which is known to produce a large amount of this polymer. Amylovoran is an acidic EPS polymer that has a pentasugar building block of one glucuronic acid and four galactose moieties. This polymer can also be modified with a pyruvate molecule that binds as a cyclic acetal (in *R* form) to the terminal galactose moiety (Fig. 6)^28,29^. Thus, the two carboxylic groups in amylovoran can react with the CF^Ò^ 350 Aminooxy dye (depicted in Fig. 6). Separately, the binding of amylovoran to the LPS may either be through a direct glycosidic linkage (possibly with a protein like EAMY_2231 that is an extracellular glycosyltranferase) or somehow through tight glycan:glycan interactions^46^. Thus, it was not surprising that addition of CF^Ò^350-labelled amylovoran to mid-log growing *E. amyolovora* D7 cells was enough to bind the labelled amylovoran to the LPS as shown by fluorescence microscopy (Fig. 2). The extracted LPS after this incubation continued to display fluorescence (Table 2). These results provide further direct support for the proposed attachment of amylovoran to the *E. amylovora* LPS, which has not been previously reported, but now opens avenues of inquiry regarding the role and exact nature of this attachment.

Phages have very high specificity for their receptor(s). Using this criterion, phages ϕEa21-4 and ϕEa46-1-A1 were used as bait to pull-down candidate protein receptors from the OMP extracts (Fig. 3). Analysis of the bound protein fractions by MS identified OmpA and flagellin as receptor proteins in OMP extracts from both *E. amylovora* and *P. agglomerans* (Table 3). Since both phages show similarity to the target receptors, further confirmation of the receptor results was carried out only on phage ϕEa21-4. Specific confirmation of the OmpA receptor results were conducted using an *E. amylovora* 1189 *waaL* mutant that had impaired LPS (lacking the O-antigen)^31^. The results indicated that LPS is not the only phage receptor, since the LPS mutants still had complete bacterial growth inhibition in the presence of both *Erwinia* phages (Fig. 4A). The absence of O-antigen provides increased accessibility to OmpA for the phages; thereby facilitating the complete growth inhibition phenotype. In complimentary parallel studies, the inclusion of an OmpA inhibitor (AOA-2 peptide) mitigated the growth inhibition effects observed by *Erwinia* phage ϕEa21-4 (Fig. 4B). Additionally, CF^Ò^350-labelled ϕEa21-4 phage was used to detect purified *E. amylovora* OmpA on a PVDF membrane (Fig. 5). In contrast to the DNA ejection studies that found only *E. amylovora* LPS was recognized, these protein studies demonstrate that both the OmpA from *E. amylovora* and *P. agglomerans* can serve as phage receptors for both ϕEa21-4 and ϕEa46-1-A1 phages. Additionally, the *E. amylovora* 1189 *waaL* infectivity results in conjunction with AOA-2 activity strongly suggest that OmpA is an important receptor for these phages. These findings are consistent with several other phages that are known to use OmpA as a receptor. For example, Sf6 phage of *Shigella flexneri* and T-even *E. coli* phages both recognize OmpA^47,48^. *Salmonella enterica* phage P22 has been noted to use both LPS and OmpA, but DNA ejection was triggered to a higher degree with OmpA than with LPS^40^. In contrast, the HK620 *E. coli* phage only uses LPS as a receptor^39^. It is worth mentioning that OmpA is one of the most abundant Gram-negative bacterial OMPs and plays different roles in virulence, biofilm formation, host infection, antibiotic resistance, and immunomodulation^49,50^. Since *P. agglomerans* does not produce amylovoran that would attach to its LPS, then it is not unexpected that phage DNA ejection did not occur in the presence of this undecorated LPS. Consequently, the sole receptor for *E. amylovora* phages on *P. agglomerans* would be OmpA. However, the variation in *E. amylovora* phages’ ability to lead to infection in *P. agglomerans* strains may still be in part due to the LPS they produce^23^. For example, *P. agglomerans* strains with different LPS structural details could lead to variation in *E. amylovora* phage accessibility to OmpA. Supporting this theory is that a similar situation was reported for *Klebsiella pneumoniae* phages^51^.

The pull-down assay with the phages indicated that flagellin is also a candidate receptor for both *E. amylovora* phages ϕEa21-4 and ϕEa46-1-A1 (Table 3). However, the presence of three different receptors for a phage is not expected and, to the best of our knowledge, has not been reported to date in the literature. Thus, it was proposed that the flagellin O-glycosylation (which can mimic LPS receptor residues) is the overriding reason that *Erwinia* phages bound flagellin in the immunoprecipitation assay^33,52^. This theory was verified when the pull-down assay was repeated with deglycosylated purified *E. amylovora* D7 FliC protein and flagellin fragments were absent from this sample. From these results, it can be concluded that flagellin is not a phage receptor, but this work serves as a caution that O-glycosylation could add confusion to receptor identification for future work with these and other phages. Interestingly, the most abundant cell surface and extracellular protein O-glycosylation is *N*-acetyl-galactosamine attached to Ser/Thr residues^53^. This overlap between LPS and flagella glycosylation is well reported in a number of bacteria, like *Helicobacter pylori*, and is a leading hypothesis to explain receptor binding by *Erwinia* phages in this study^33^.

In conclusion, the results presented herein provide clear evidence that the *E. amylovora* phages, ϕEa21-4 and ϕ Ea46-1-1A, have two receptors on *E. amylovora* cells (OmpA and LPS) and only one receptor on *P. agglomerans* (OmpA). The results also indicate for the first time that there is a tight association of amylovoran to *E. amylovora* LPS. The possibility that this is an *N*-acetyl-galactosamine-mediated glycosidic linkage between amylovoran and LPS is supported by enrichment of these sugars in phage treated LPS samples, however, further confirmation is needed. Previous publications that mention amylovoran as a receptor for *E. amylovora* had difficulty in explaining how amylovoran, a secreted EPS that was thought to be loosely associated with the cell surface, could serve as an efficient receptor for the cells^16^. In this study, evidence that amylovoran is attached to the LPS, or at least tightly associated with *E*. *amylovora*, could help clarify this scenario. Indeed, this modified view coincides well with previous mutational work that notes changes in LPS can lead to phage resistance in *E. amylovora.* With this combined data in mind, we propose a new model for the cell surface arrangement and phage infection of *E. amylovora* (Fig. 6). In this model, the amylovoran is proposed to be attached to the LPS through an *N*-acetyl-galactosamine residue, which may either be one of the amylovoran four galactose moieties or found already on the LPS (Fig. 6). *E. amylovora* phages recognize this attachment between amylovoran and LPS (as amylovoran is very characteristic to *E. amylovora*) and degrade this linkage with tail-spike enzyme activity to have access to OmpA. This model also provides a viable explanation for the role of the EAMY_2231 mutation in the Y2 phage resistance that was previously published^54^. Upon analysis, this mutant demonstrated low amylovoran production but also altered LPS structure and low growth in LB^54^. The encoded EAMY_2231 protein (accession #: D4HW82) has 47 and 24% amino acid similarities to the *Thelohanellus kitauei* putative glycosyltransferase EpsJ (accession #: A0A0C2MC88) and *Campylobacter jejuni* PglA (accession #: Q0P9C9), respectively (Supplementary data; Figure S3 and Table S2). Both PglA and EpsJ aid in transferring *N*-acetyl-galactosamine to a lipid carrier for its subsequent translocation and *N*-glycosylation. This activity in EAMY_2231 may be reflected in amylovoran binding to the LPS by somehow aiding *N*-acetyl-galactosamine linkage and directly account for Y2 phage resistance if this is lost. While plausible, further confirmation of this model is necessary to fully exploit this knowledge in phage development for PCS applications from an LPS receptor standpoint. In the meantime, our increased understanding of the role of OmpA now allows us to directly understand how phages target and infect both *E. amylovora* and carrier bacteria *P. agglomerans*, and the dynamics of the PCS system.

## Methods

### Bacteria and bacteriophages isolates

All bacterial strains used in this study are listed in Table 5. Cultures were stored at − 80 °C in Microbank cryobeads (Pro-Bank Diagnostics, Richmond Hill, ON, Canada). To prepare the working culture stock, one Microbank cryobead was mixed with one drop of phosphate-buffered saline (PBS) (10 mM, pH 7.2) and plated on 2.3% (w/v) Difco™ nutrient agar (NA) plates (BD, Sparks, MD, USA). The plates were incubated for 16 to 18 h at 27 °C and then stored at 4 °C for 1 to 2 wks. Working cultures were obtained from the initial cultures by streaking single colonies onto NA and incubating at 27 °C for 16 to 18 h.

**Table 5:** Bacterial strains used in this study.

Strain	NCBI GenBank accession #	References
*Pantoea agglomerans*		
39-7	JACSWZ000000000	^55^
*Erwinia amylovora*		
6-4	JAAEVD000000000	^56^
D7	JAAEUT000000000	^56^

The two lytic *E. amylovora* bacteriophages used in this study were ϕEa21-4 (myovirus) and ϕEa46-1-A1 (podovirus) as listed in Table 6. To propagate each phage, a bacterial host suspension was prepared by suspending 5–6 colonies in 3 mL of 0.8% (w/v) nutrient broth (NB) (BD, Sparks, MD, USA) to obtain an OD_600_ of ~ 0.6. Using a 250-mL baffled Erlenmeyer flask, 100 μL of the bacterial suspension was added to 75 mL of NB, which was then incubated at 27 °C with 150 rpm shaking (New Brunswick Innova., Eppendorf, Hamburg, Germany) for 3 to 4 h. A 100 μL aliquot of phage stock (5.0 × 10^9^ PFU/mL) was added and the mixture was incubated for 16 to 18 h at 27 °C with 150 rpm shaking. Following incubation, 1 mL of chloroform was added to the culture and incubated with shaking for 5 min. The bacterial culture was subjected to centrifugation at 8500*×g* at 4 °C for 15 min, the pellet discarded, and the supernatant filtered through a 0.22 μm Steriflip filter (Millipore, Burlington, MA, USA). The working phage stocks were stored at 4 °C in dark amber glass vials until needed. Bacterial and phage enumeration was carried out using a previously published qPCR protocol^23^.

**Table 6:** *E. amylovora* phages used in this study.

Phage	Species	NCBI GenBank accession number	*E. amylovora* Host	References
ϕEa21-4	*Kolesnikvirus* Ea214	NC_011811.1	6-4	^57^
ϕEa46-1-A1	–	N/V	D7	^57^

### LPS purification

LPS extraction from *E. amylovora* and *P. agglomerans* was carried out using previously published methods^36,37^. Briefly, 1 mL of bacterial overnight cultures were pelleted and washed once with PBS. Cell pellets were resuspended in Hitchcock and Brown lysis buffer (250-mL; 1.5 M Tris–HCl, pH 6.8, 50% (v/v) Glycerol and 10% (w/v) SDS) and heated in a boiling water bath for 30 min. After cooling, 2 mL of DNase I (20 mg/mL) was added, and incubated at 37 °C for 30 min. A 2 mL volume of Proteinase K (20 mg/mL) was then added, and samples were incubated for 16 to 18 h at 55 °C. Extracted LPS was pooled and precipitated by adding sodium acetate to a final concentration of 0.5 M and 9 to 10 volumes of 95% (v/v) ethanol. After incubation for 16 to 18 h at -20 °C, the LPS was collected by centrifugation at 10,000×*g* at 4 °C for 20 min. LPS was then dissolved in distilled water and kept at -20 °C until needed.

### Fluorescence DNA ejection assay

LPS samples of four biological replicates of *E. amylovora* D7 and *P. agglomerans* 39–7 were extracted by the abovementioned method. LPS concentrations were determined using the PyroGene®_recombinant factor C (Endotoxin detection assay) (Lonza, MD, USA). Following concentration determination, equal amounts of LPS samples were added in a plate in three replicates of each sample with 1.1 mM Yo-Pro-1 Iodide (Invitrogen™), then equilibrated at 37 °C in PBS pH 7.4 buffer before excitation of the sample at 491 nm and detection at 509 nm as a baseline reading. After the addition of phages to a final concentration of ~ 1.0 × 10^8^ PFU/mL, ejection of DNA in the presence of LPS was followed for 4 h by taking readings at 5 min intervals^38,39^. Relative fluorescence percentage was calculated using the equation: ((F_t_ − F_0_)/(F_max_ − F_min_)) × 100, then plotted versus the time.

### Analysis of phage-treated LPS

*E. amylovora* D7 LPS was extracted as mentioned above, then divided into two fractions of 50 mg each. One of the samples was treated by incubation for 16 to 18 h at room temperature with purified phages ϕEa21-4 (0.3 mL of 3.6 × 10^9^ PFU/mL in dH_2_O) and denoted as the phage-treated (T) sample, while the other control (C) sample only had 0.3 mL of dH_2_O added. After incubation, LPS samples were precipitated and sent for detailed carbohydrate analysis at the GlycoNet Integrated Service (University of Alberta). Briefly, samples were hydrolyzed with 1% (v/v) trifluoro-acetic acid diluted in H_2_O, lyophilized, and resuspended in acetic acid and dimethylsulfoxide (3:7, v/v). Hydrolyzed fragments were labelled with sodium cyanoborohydride and 2-aminobenzamide, cleaned using LudgerClean SPE, lyophilized and then resuspended in aqueous acetonitrile (60%, v/v) for chromatographic analysis. 2-AB-labeled glycans were analyzed by HPLC using Waters ACQUITY UPLC BEH Amide column (130 Å, 1.7 μm, 2.1 × 150 mm). A gradient elution of 85 to 50% mobile phase B was executed over 15 min at 0.5 mL/min, 45 °C (mobile phase A = 50-mM ammonium formate pH 4.4, mobile phase B = acetonitrile, 100%). Electrospray ionization-mass spectrometry (ESI–MS) detection was used in positive mode on a ThermoFisher Orbitrap Exploris 240 with acquisition of MS^2^ fragment spectra. When accompanying fluorescence detection (FLD) was determined, an excitation wavelength of 320 nm and an emission of 420 nm were used^35^. Data represented as glycan fragments depleted or enriched in the phage-treated LPS sample relative to the control LPS.

### Amylovoran purification

Amylovoran was purified as previously published^28,29^. Briefly, *E. amylovora* D7 cells were grown for 24 h at 28 °C in M9 liquid media supplemented with 0.2% (w/v) glucose and 0.1% (w/v) yeast extract. Cells were removed by centrifugation, then tricholoroacetic acid was added to the supernatant to a final concentration of 10% (v/v) for 30 min with incubation on a shaker, and the supernatant was cleared by centrifugation at 8200*×g* for 10 min, 4 °C. Amylovoran was precipitated with cetylpyridinium chloride (1% v/v final concentration). Precipitate was resuspended in PBS and amylovoran was recovered by precipitation with 2:1 (v/v) of cold absolute methanol and overnight incubation at − 20 °C to get rid of the cetylpyridinium chloride. Amylovoran was collected by centrifugation at 8200*×g* for 20 min at 4 °C and then resuspended in deionized water and dialysed extensively (12 kDa cut-off) and lyophilized.

### Fluorescence labelling

According to the manufacturing protocol, CF® dye was prepared as a 5 mM stock solution in water, and amylovoran or OmpA stock solution was prepared as 20–100 mM concentrations in PBS. CF® dye was added in 50 molar equivalents to the amylovoran/OmpA stock solution. The ligation reaction was initiated by adding a 1/10 volume of aniline acetate, vortexing and incubating at room temperature for 5 to 10 h in the dark. Free dye was removed by dialysis twice in 2 L PBS at 4 °C.

### Outer membrane protein purification

OMP extraction from *E. amylovora* and *P. agglomerans* was carried out according to a previously published method^59^. Briefly, bacterial overnight cultures were pelleted and washed once in PBS. Cell pellets were resuspende d in lysis buffer (20 mM Tris–HCl, pH 7.0, 100 mM NaCl, 5 mM EDTA, 80 mg/mL DNase I, 80 mg/mL RNase A, 300 mg/mL lysozyme, and protease inhibitor cocktail) and then subjected to two rounds of liquid N_2_/RT freeze–thaw before sonication for 2 min (30 s on/30 s off). Samples were then diluted by an equal volume of MilliQ H_2_O, then clarified by subjecting them to centrifugation at 8200*×g* (Beckman JA25.5 rotor, 10,000 rpm) for 20 min at 4 °C. Total membrane proteins were collected from the filtrate by ultracentrifugation at 117,700*×g* (Beckman Ti70, 40,000 rpm) for 1 h at 4 °C. Membrane protein pellets were then resuspended in 30 mM Tris–HCl, pH 8.0, and loaded onto a sucrose step gradient that consisted of three sucrose layers from 30, 50 and 70% (v/v) sucrose solutions and subjected to ultracentrifugation at 125,700*×g* (Beckman SW32, 32,000 rpm) for 20 h at 4 °C. The OMP extract was collected from the colored band between 50 and 70% (w/v) sucrose bands.

### Phage biotinylation and immunoprecipitation assay

The steps for the phage biotinylating and immunoprecipitation assay are illustrated in Fig. 3. Briefly, (1) phages were collected from the phage filtrate (12 mL of ~ 10^9^ PFU/mL) by ultracentrifugation at 7350*×g* (Beckman Ti70, 10,000 rpm) for 18 h at 4 °C. (2) The pellet was then dissolved in PBS and biotinylated using EZ-link Sulfo-NHS-LC-LC-Biotin (Thermo Scientific, Rockford, IL, USA) according to the manufacturer’s instructions. Unreacted EZ-link Sulfo-NHS-LC-LC-Biotin was dialyzed using a Slide-A-Layzer Dialysis Cassette (MWCO 3500; Thermo Scientific, Rockford, IL, USA) for 2 h in PBS. (3) Biotinylated phage was then conjugated with Ultra HBC Streptavidin agarose resin, (GoldBio, MO, USA) according to the manufacturer’s protocol to form Phage-Biotin-agarose complexes that were used as a bait for receptor identification in the OMP extracts. (4) These OMP extracts were incubated with Phage-Biotin-agarose beads for 1 h with mild shaking at 4 °C and beads were subsequently washed twice using PBS buffer (with 1% (v/v) protease inhibitor cocktail). (5) On-bead digestion was carried out according to the previously published protocol^28^. (6) The pulled-down proteins were identified by MS/MS spectrometry and data was analyzed using the MaxQuant software. The immunoprecipitation assay was repeated with O-deglycosylated *E. amylovora* D7 FliC protein and compared with the native (glycosylated) one. The O-deglycosylation reactions were conducted according to the manufacturer’s protocol for the non-denature standard protocol (Glycoprotein Deglycosylation Kit, Calbiochem®, Cat. #: 362280). Each reaction was 50 μL as follow: 38 μL of *E. amylovora* D7 FliC (3–5 mg/mL in deionized water), 10 μL 5X Reaction Buffer, 1 μL of β1,4-galactosidase and 1μL β-*N*-acetyl-glucosaminidase. Reactions were incubated for 3–5 days at 37°C. Four replicates were prepared for the O-deglycosylation reaction and 3 replicates as controls (no enzymes were added).

### OmpA candidate receptor confirmation

*E. amylovora* 1189 wildtype and the *waaL* mutant were kindly provided by Dr. G. Sundin (Michigan State University, USA). Bacterial cultures (1.0 × 10^3^ CFU/mL) were incubated with ϕEa21-4 or ϕEa46-1-A1 at MOI of 1.0. The effect of phages on bacterial growth was monitored in triplicate for 24 h by monitoring the changes in OD_600_ using a plate reader while incubated at 27 °C, where the changes in turbidity represent changes of the bacterial growth due to the phage infection^31^. II: The effect of AOA-2 peptide (cyclic peptide: &Trp-D-Pro-Arg-Trp-DPro-Arg&, a known OmpA inhibitor, with final concentrations of 125 and 250 mg/mL) presence on the infection of *E. amylovora* D7 by ϕEa21-4 was tested compared to the control^32^. III: OmpA was partially purified from the *E. amylovora* strain D7 OMP extract using a phenyl-sepharose column (colume 20 mL) using a gradient of Buffer A (15 mM PBS, pH 7.4, with 1.0 M Ammonium Sulfate) from 100 to 0% with 5 column volumes and Buffer B was (15 mM PBS. PH7.4)^58^. Fractions with proteins of the expected *E. amylovora* OmpA molecular weight were tested for phage ϕEa21-4 inactivation by mixing with the phage lysate (1:1, v/v) then tested by plaque formation by soft agar overlay assay. The fraction with the strongest phage inactivation was considered as partially purified OmpA. The partially purified OmpA was electrophoresed on a 12% Native PAGE, blotted on Immobilon-P membrane, and detected with CF350-labelled ϕEa21-4 phage. After membrane incubation with the labelled phage and washing with PBS, the membrane was imaged using Molecular Imager® Gel Doc® XR (Bio-Rad, USA).

### SDS-PAGE and protein blotting

Protein samples (~ 20 mg) were mixed with SDS-PAGE sample buffer (90 mM Tris-base, pH 6.8, 2% (w/v) SDS, 0.02% (w/v) Bromophenol Blue, 20% (v/v) glycerol in dH_2_O) and boiled for 5 min at 95°C, then cooled to room temperature. These samples were then loaded on a 1 mm 12% (w/v) resolving gel and run for 90 min at 120 V in Mini-PROTEAN system (Bio-Rad, USA). Protein transfer for Western blotting was performed using an Immobilon-P transfer membrane (Millipore, USA) and run for 60 min at 100 V. Gels and membranes were imaged using Molecular Imager® Gel Doc® XR (Bio-Rad, USA).

## Supplementary Information

Below is the link to the electronic supplementary material.

Supplementary Material 1

Supplementary Material 2

Supplementary Material 3

## Data Availability

No datasets were generated or analysed during the current study.
