# Supplementary material for: Phage host interactions reveal LPS and OmpA as receptors for two Erwinia amylovora phages
Source: Sci Rep. 2025 Oct 21;15:36527. doi: 10.1038/s41598-025-15724-z (PMC12540986; doi:10.1038/s41598-025-15724-z)
Supplement: Supplementary file 1 — Supplementary Material 1 [file 41598_2025_15724_MOESM1_ESM.pdf]

Table S1. Glycan peaks (m/z, H^+) and Relative / Normalized Areas

tR (min)	m/z H+	ID (?)	Treated	Control	T/C %
2.79	271.14	Pen	3.22	4.77	67.60
4.10	301.14	Hex	7.49	7.73	96.89
4.38	301.14	Hex	26.45	24.05	109.99
5.77	331.15	Hep	3.71	3.52	105.40
6.49	331.15	Hep	1.47	1.61	91.16
6.96	445.18	?	1.40	1.12	124.79
7.98	371.14	PyrHex	5.76	4.93	116.93
8.16	371.14	PyrHex	1.51	1.42	106.98
9.69	463.19	Hex2 or PenHep	4.93	4.77	103.22
9.69	425.21	?	4.93	4.77	103.22
10.48	504.22	HexHexNAc	0.81	0.61	132.58
10.73	463.19	Hex2	15.45	16.07	96.08
11.28	463.19	Hex2 or PenHep	1.04	0.97	106.33
11.52	463.19	Hex2 or PenHep	1.28	1.12	114.03
12.26	609.24	Hex2dHex	9.84	8.58	114.68
14.06	609.24	Hex2dHex	3.02	2.49	121.41
14.06	523.22	Hep2	3.02	2.49	121.41
14.36	625.24	Hex3	1.80	1.76	102.19
16.00	755.31	Hex2dHex2	2.14	1.88	113.88

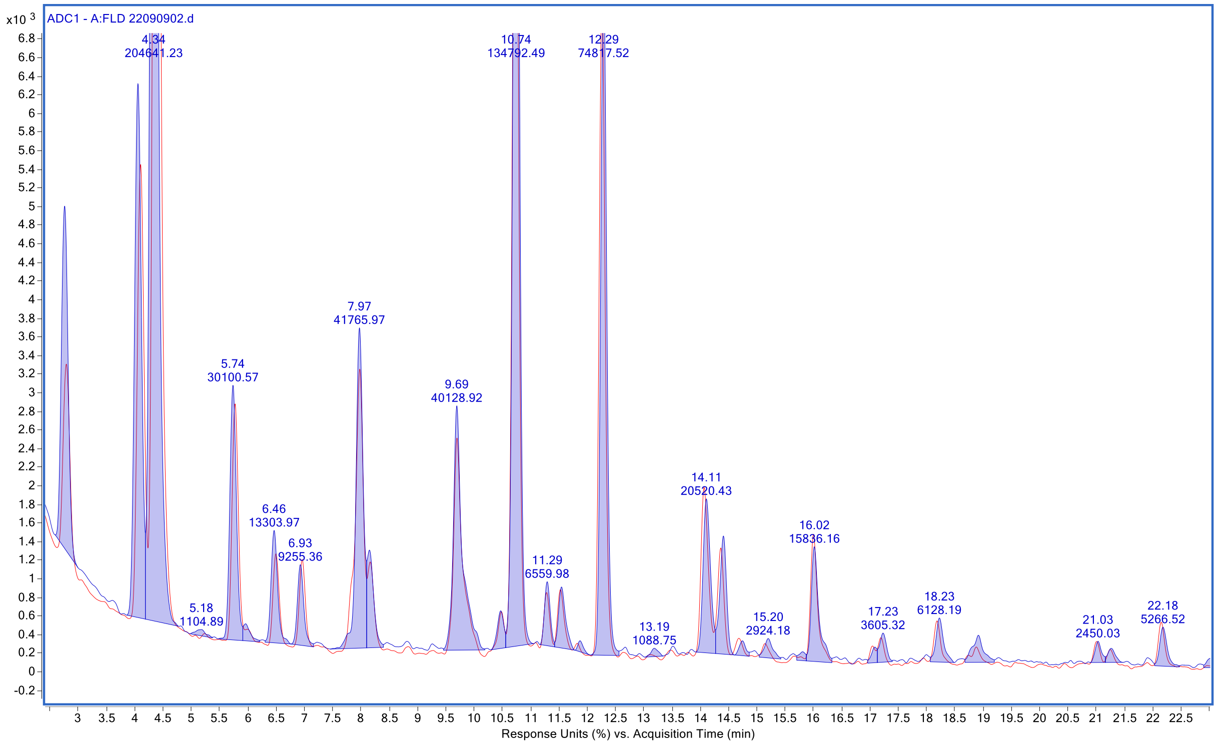

Figure S1. Chromatogram of normalized (% Maximum Area of Each Sample) fluorescence intensity of separated glycan fragments.

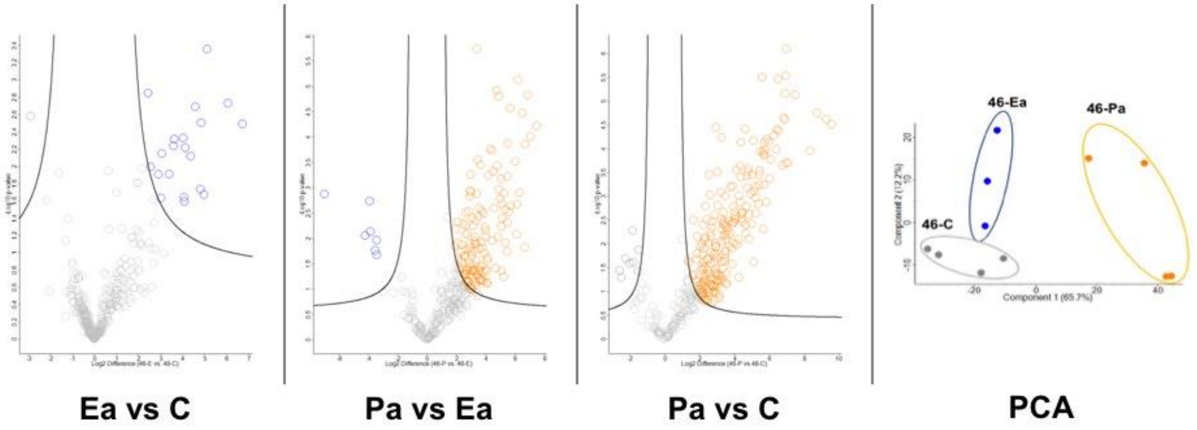

Figure S2. Analysis of pulldown assay with ϕEa46-1-A1. Volcan plots showed the results of OMPs extracts of *E. amylovora* vs control (Ea vs C), OMPs extract of *Pantoea agglomerans* vs that of *E. amylovora* (Pa vs Ea), and OMPs extract of *Pantoea agglomerans* vs control (Pa vs C). PCA graph showed the principal component analysis for the whole experiment.

```
CLUSTAL O(1.2.4) multiple sequence alignment


PglA        -MRIGFLSHAGASIYHFRMPIIK-AL--KDRKDEVFVIV--PQDEYTQKLRDLGLKVIVY      54
EpsJ        MKKLCYFI---NSDWYFDLHWIDRAIAARDAGYEIHIISHFVDGKVLNRFKTFGFKCHNV      57
D4HW82      MHKICYFV---NTDWYFDLHWLERALSARDNGYEVHIICNFDDEIIYEKLKGLGLKCYKL      57
              :: ::     : ::* :  :. *:  :*   *:.:*    :    :::: :*:*

PglA        EFSRASLNPFVVLKNFFYLAKVLKNLNLDFIQSAAHKSNTFGILAAKWAKIPYRFALVEG      114
EpsJ        PLDAQSFNALTFFRAFFASRRIIKEINPDLLHCITIKPCLIGGLLSRANNRPVIVSF-VG      116
D4HW82      NVHSQSLSVIRFTRSVYKFFSILKSISPDILHCITIKPIIIGGLYSFYKKKPVVLSF-VG      116
             .   *:. : . : .:    ::*.:. *:::. : *   :* * :   : *  .::  *

PglA        LGSFYIDQGFKANLVRFVINSLYKLSFKFAHQ-FIFVNESNAE-FMRNLGLKENKICVIK      172
EpsJ        LGRVFSCDTGLLKLLRNITIMAYKYIASNKRSIFMFEHDKDRKKLAGFVGIDYQQTIVID      176
D4HW82      LGRLFDGETTLLKILKYFITKIYKVIFKNPKALFIFEHNSDRERLLKLTHGPIHQTEVIE      176
            ** .:  :    :::: .    **   .  :  *:* ::.: : :        ::  **.

PglA        SVGINLKKFFPIYVESEKKELFWKNLNIDKKPIVLMIARALWHKGVKEFYESATMLKDKA      232
EpsJ        GAGINPDIY------------KYSLEQERDVPVVLFASRMLWSKGLGDLIEAKKILESRN      224
D4HW82      GAGVNTDLF------------RYQPEPVNEIPIILFASRLIWSKGLYDLIQAKLKLKDQD      224
            ..*:* . :             :.     . *::*: :* :* **: :: ::   *:.:

PglA        NF--------VLVGGRDENPSCASLEFLNSGAVHYLGARSDIVELLQNCDIFVLPS-YKE      283
EpsJ        IHFTLNVAGILVENDKDAISLAVIQQWHKEGLINWLGHSSNVCDLIEESNIVALPSIYSE      284
D4HW82      VHFRILVAGITVTDDKDAIKDSVIEKWVENGWIEWLGKSDDVARLISGANLVVLPSVYNE      284
             .         : ..:*    ..  :: :.* :.:**  .::  *:. .::..*** *.*

PglA        GFPVSVLEAKACGKAIVVSDCEGCVEAISNAYDGLWAKTKNAKDLSEKISLLLEDEKLRL      343
EpsJ        GVPRILLEASSVGRACIAYDVGGCDSLIINNDNGIIVKSNSPQELADKLEFLLANPKARV      344
D4HW82      GIPRILLESCAIGRACVCYDSGGCGSLVIDGENGFLVSKHDIDSLAERIGYLLQYPSTRI      344
            *.*  :**: : *:* :  *  ** . : :  :*: ...:. ..*::::  **   . *:

PglA        NLAKNAAQDA-LQYDENIIAQRYLKLYDRVIKNV  376
EpsJ        EMGIKGRQRVQDKFSSGMIISKTLKTYHDVVQG-  377
D4HW82      KMGRRGTEIISEKFSSEIVIASTLAAYNELI---  375
            ::. .. :    ::.. ::    *  *. ::
```

Figure S3. Sequence alignment of ORF of the Eamy_2231 gene. ORF of the Eamy_2231 gene (accession #: D4HW82) was aligned with the sequences of N,N'-diacetyl-bacillosaminyl-diphospho-undecaprenol alpha-1,3-N- acetylgalactosaminyltransferase, PglA (accession #: Q0P9C9)**,** from *Campylobacter jejuni* as a part of bacterial N-linked glycosylation pathway and putative glycosyltransferase EpsJ (accession #: A0A0C2MC88) *Thelohanellus kitauei.*

Table S2. Similarities between Eamy_2231 ORF (accession #: D4HW82) and two galactosyltransferases, PglA and EpsJ.

	PglA	EpsJ	D4HW82
**PglA**	**100**	23.12	24.09
**EpsJ**	23.12	**100**	47.2
**D4HW82**	24.09	47.2	**100**
