# Supplementary material for: Phage host interactions reveal LPS and OmpA as receptors for two Erwinia amylovora phages
Source: Sci Rep. 2025 Oct 21;15:36527. doi: 10.1038/s41598-025-15724-z (PMC12540986; doi:10.1038/s41598-025-15724-z)

Original Files

Figure 2

Trans image

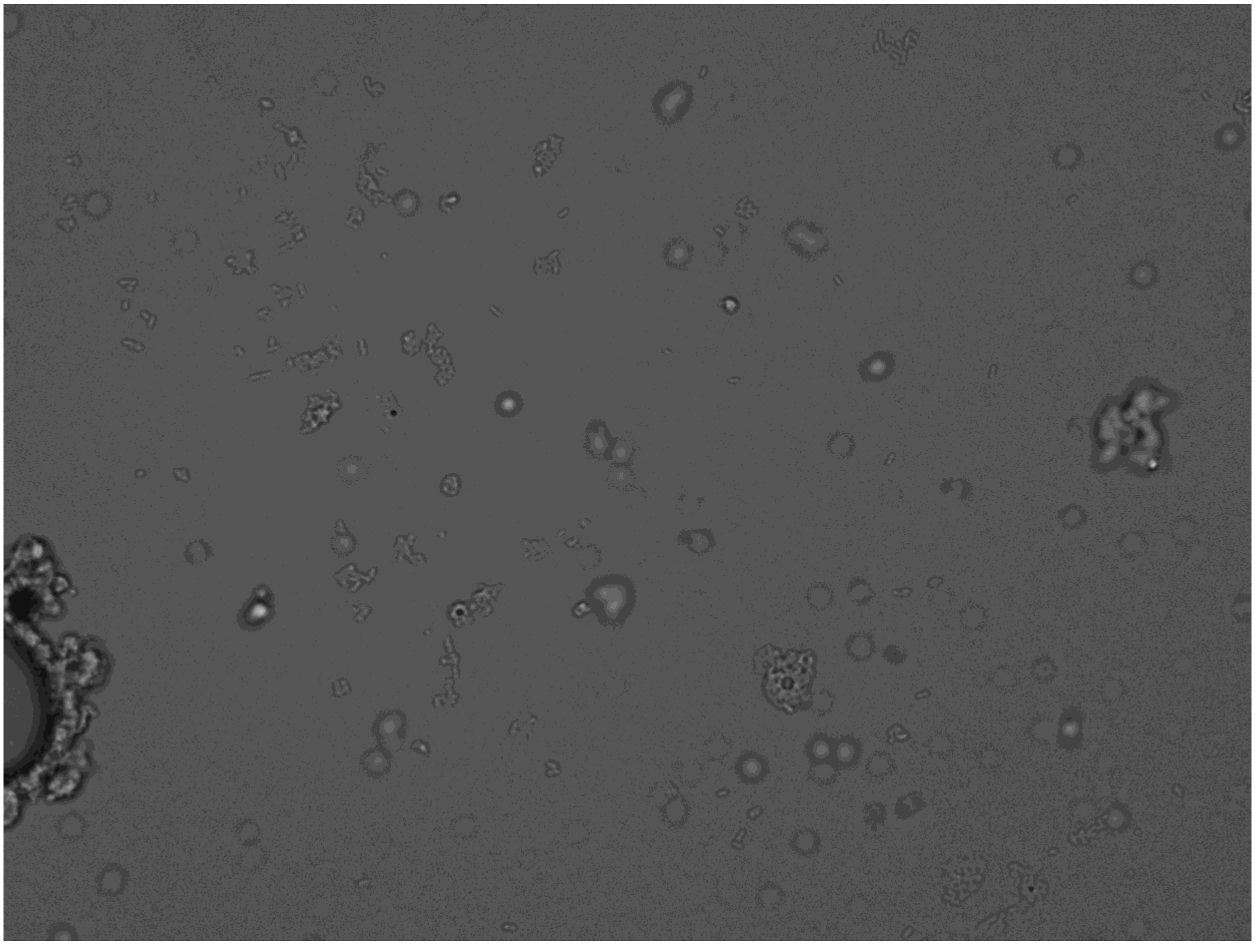

Superimposed image

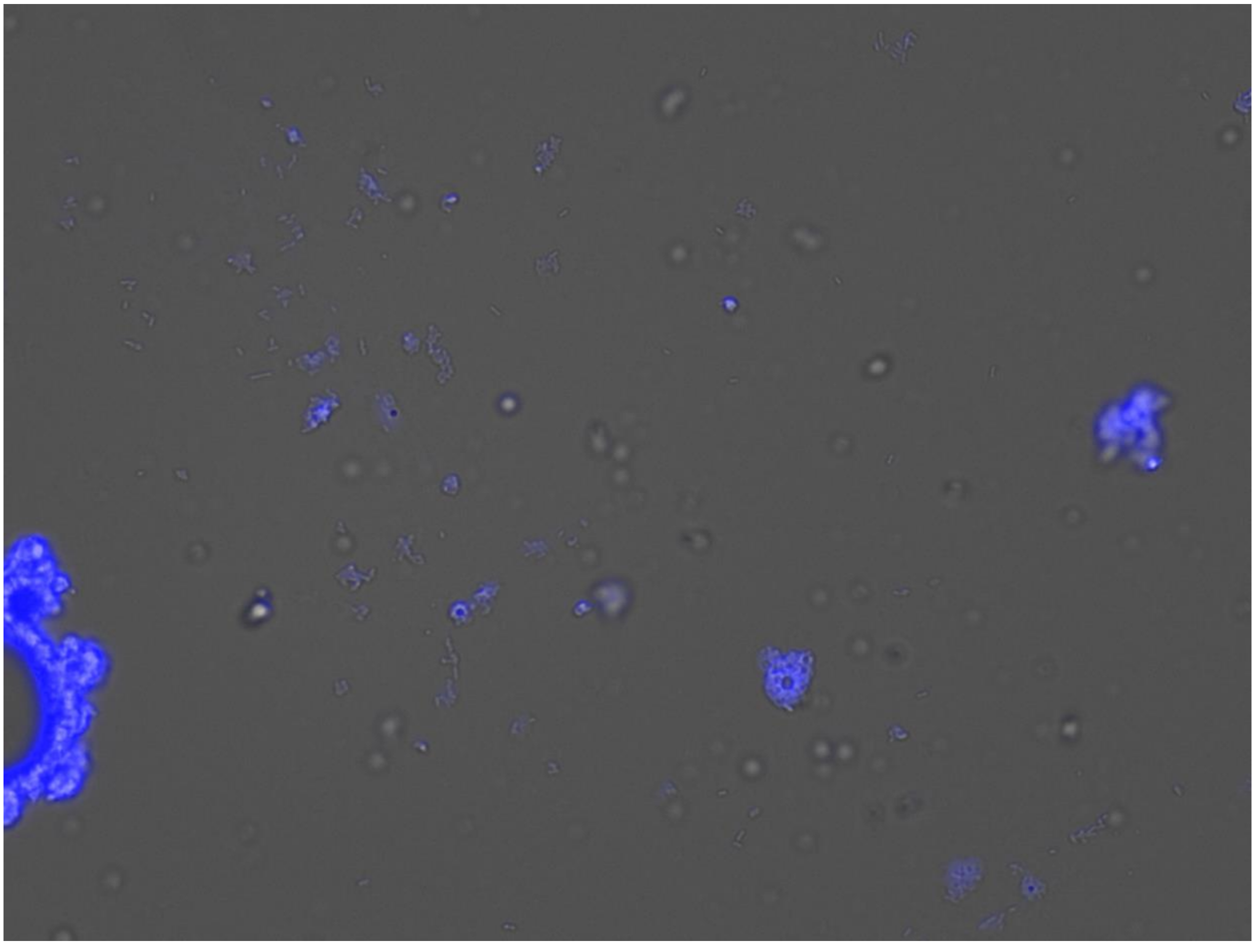

DAPI imaging

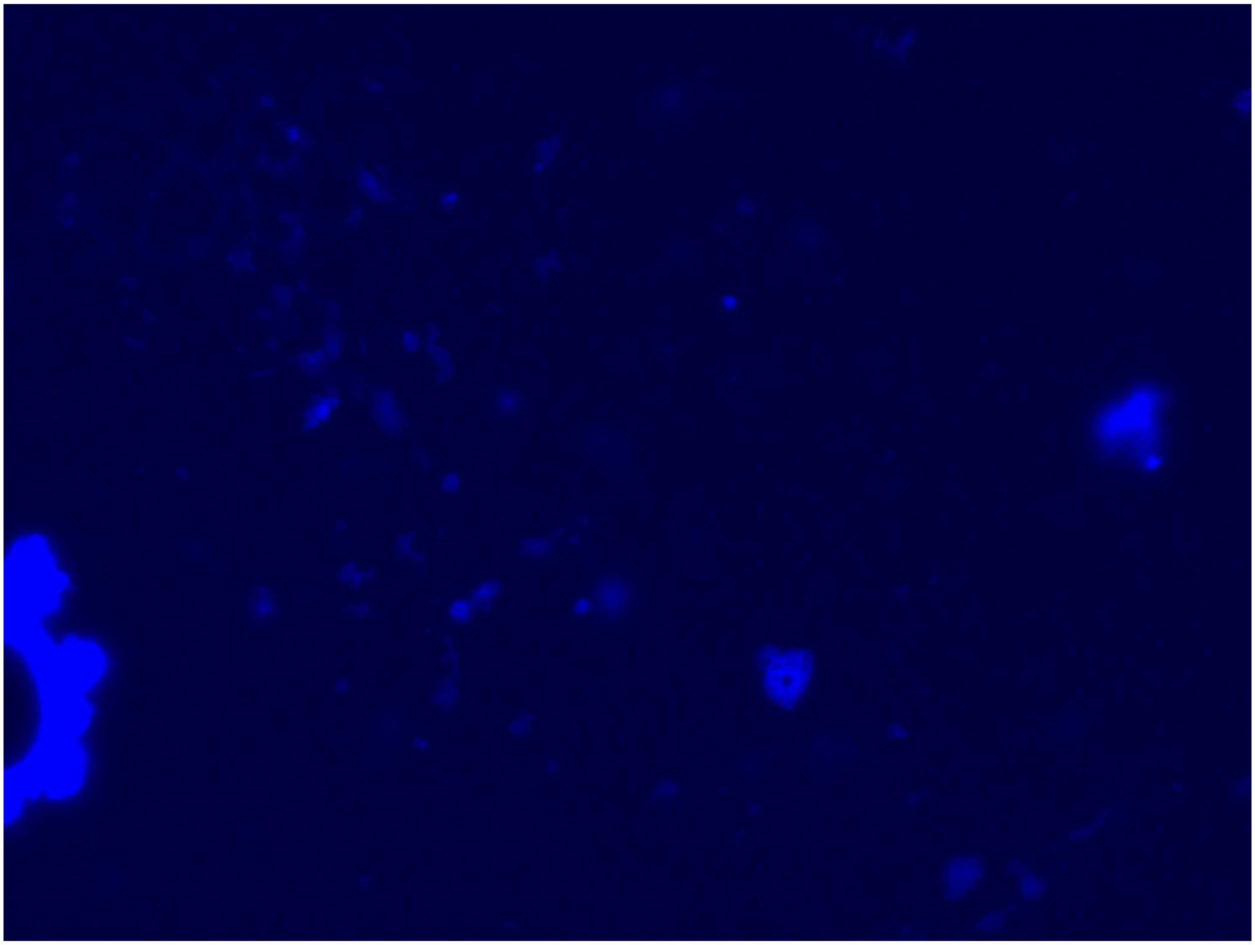

Figure 5

UV Transmitter after incubation with fluorescent-labelled phage and then washing

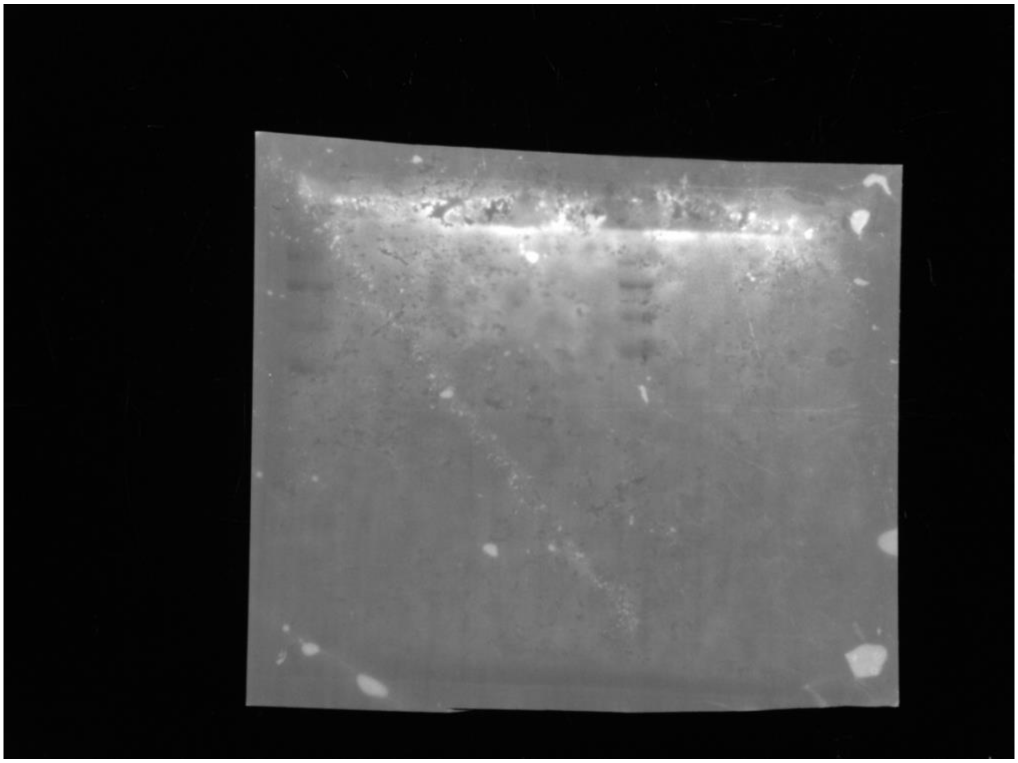

UV Transmitter before incubation with fluorescent-labelled phage

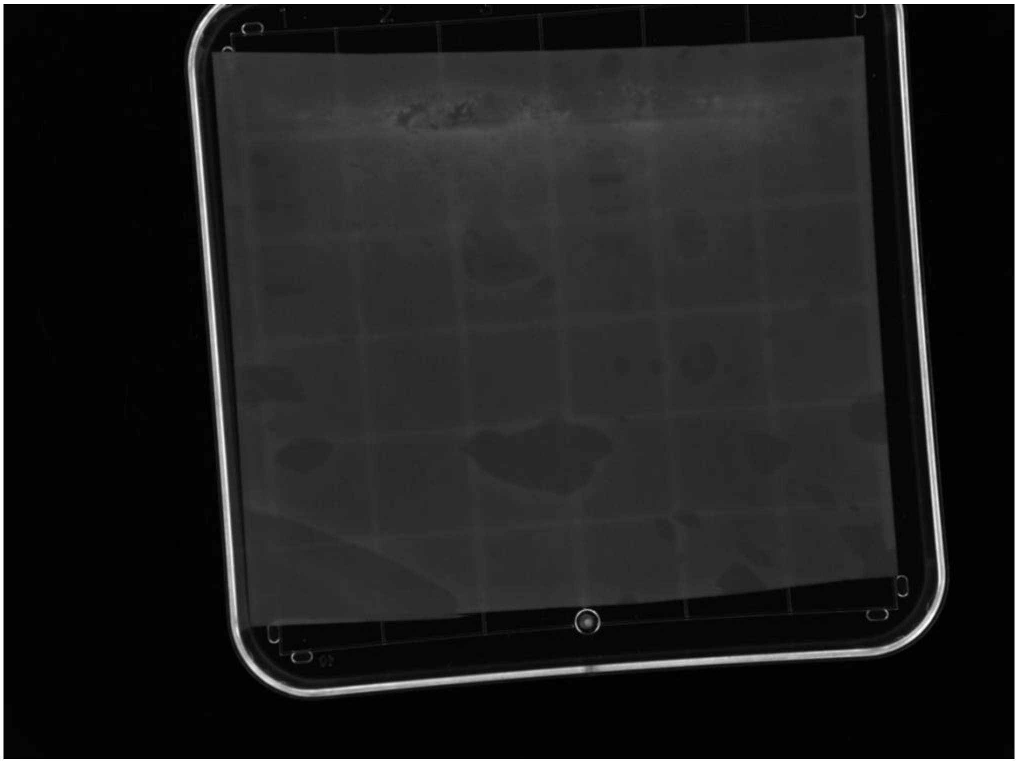

Light imaging

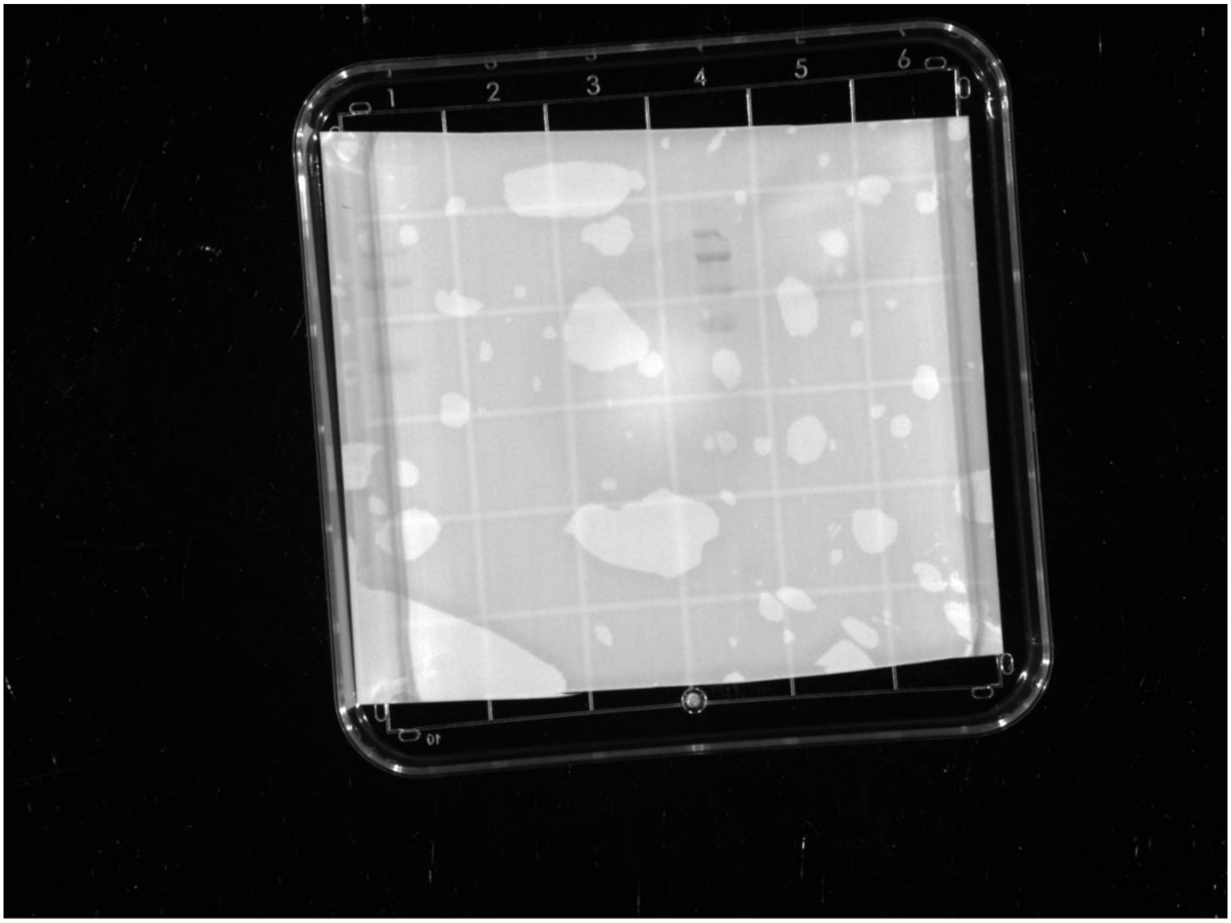

Supplement: Supplementary file 3 — Supplementary Material 3 [file 41598_2025_15724_MOESM3_ESM.pdf]
